# Economic burden of locoregional and metastatic relapses in resectable early-stage non-small cell lung cancer in Spain

**DOI:** 10.1186/s12890-023-02356-0

**Published:** 2023-02-21

**Authors:** Javier De Castro, Amelia Insa, Roberto Collado-Borrell, Vicente Escudero-Vilaplana, Alex Martínez, Elena Fernandez, Ivana Sullivan, Natalia Arrabal, David Carcedo, Alba Manzaneque

**Affiliations:** 1 Hospital Universitario La Paz, Madrid, Spain; 2 Hospital Clínico Universitario de Valencia, Valencia, Spain; 3 Hospital General Universitario Gregorio Marañon, Madrid, Spain; 4 Hospital Universitari Vall d’Hebron, Barcelona, Spain; 5 OSI Bilbao-Basurto, Bilbao, Spain; 6 Hospital de la Santa Creu i Sant Pau, Barcelona, Spain; 7 Roche Farma S.A., Madrid, Spain; 8 Hygeia Consulting, Madrid, Spain; 9 Hospital Universitari Mútua Terrassa, Barcelona, Spain

**Keywords:** Economic burden, Early-stage NSCLC, Locorregional relapse, Metastatic relapse

## Abstract

**Background:**

There are scarce data of the costs of non-small cell lung cancer (NSCLC) recurrence in Spain. The objective of this study is to assess the economic burden of disease recurrence, for both locoregional and/or metastatic relapses, after appropriate early-stage NSCLC treatment in Spain.

**Materials and methods:**

A two-round consensus panel of Spanish oncologists and hospital pharmacists was conducted to collect information on patient’s flow, treatments, use of healthcare resources and sick leaves in patients with relapsed NSCLC. A decision-tree model was developed to calculate the economic burden of disease recurrence after appropriate early-stage NSCLC. Both direct and indirect costs were considered. Direct costs included drug acquisition and healthcare resources costs. Indirect costs were estimated using the human-capital approach. Unit costs were obtained from national databases (euros of 2022). A multi-way sensitivity analysis was performed to provide a range to the mean values.

**Results:**

Among a cohort of 100 patients with relapsed NSCLC, 45 patients would have locoregional relapse (36.3 would eventually progress to metastasis and 8.7 would be considered in remission) and 55 patients would have metastatic relapse. Over time, 91.3 patients would experience a metastatic relapse (55 as first relapse and 36.6 after previous locoregional relapse). The overall cost incurred by the 100-patients cohort is €10,095,846 (€9,336,782 direct costs, €795,064 indirect costs). The average cost of a locoregional relapse is €25,194 (€19,658 direct costs, €5536 indirect costs), while the average cost a patient with metastasis who receives up to 4 lines of treatment is €127,167 (€117,328 direct, €9839 indirect).

**Conclusions:**

To our knowledge, this is the first study that specifically quantifies the cost of relapse in NSCLC in Spain. Our findings shown that the overall cost of a relapse after appropriate treatment of early-stage NSCLC patients is substantial, and it increases considerably in the metastatic relapse setting, mainly due to the high cost and long duration of first-line treatments.

**Supplementary Information:**

The online version contains supplementary material available at 10.1186/s12890-023-02356-0.

## Background

Lung cancer is the leading cause of cancer-related death worldwide [1]. The Spanish Society of Medical Oncology (SEOM) estimated 30.948 new cases by 2022 in Spain [2]. Also, in 2020 the World Health Organization’s (WHO) Global Observatory on Cancer (GLOBOCAN) reported 1.8 million deaths worldwide [3]. In addition, the Surveillance, epidemiology, and end results (SEER) Program reports 5-year relative survival of 21.7% for 2011–2017 period [4].

Non-small cell lung cancer (NSCLC) is the most common histological type and accounts for 85–90% of all lung cancers [5, 6]. Nearly 60% of NSCLC patients are diagnosed with advanced-stage disease (stage III-B or IV) as opposed to patients diagnosed at an early stage (stage I -III-A, localized [18%] regional [22%]), when the tumor can be treated by surgical resection. [4, 7, 8]. Patients with stage IV are not suitable for surgical resection; however, they are usually candidates for clinical trials, palliative treatment and/or systemic therapy, including chemotherapy, targeted therapy, or immunotherapy, depending on the histological subtype, performance status (PS) and results from biomarker testing [9].

In advanced disease, the treatment with tyrosine kinase inhibitors (TKIs) is the main approach to target the majority of NSCLC driver genetic alterations including: the anaplastic lymphoma kinase gene (*ALK*), the proto-oncogene tyrosine-protein kinase 1 (*ROS1*) and the epidermal growth factor receptor (*EGFR*) [1, 9–12]. Moreover, several human immune-checkpoint–inhibitor antibodies are available to inhibit the programmed-death 1 receptor (PD-1) or the PD-1 ligand (PD-L1), improving antitumor immunity [9]. Other emerging biomarkers are the human epidermal growth factor receptor 2 (HER2), v-raf murine sarcoma viral homolog B1 (*BRAF*), the rearranged during transfection (*RET)* gene fusions and the mesenchymal-epithelial transition factor (*MET*) [1, 9, 11, 12].

In patients with early-stage NSCLC and with no medical contraindications to surgery, surgical resection remains the treatment of choice [13, 14]. However, even after resection, 30–55% patients will develop disease recurrence within the first 5 years of surgery [13, 15], which is the main cause of mortality during postresectional treatment of NSCLC [16].

Many trials have been conducted to improve the survival of early-stage NSCLC patients and some have demonstrated the benefits of adjuvant chemotherapy or immunotherapy [17]. For instance, in the PACIFIC trial, durvalumab following concurrent chemoradiotherapy was associated with significant improvements in the overall survival (OS) and progression-free survival (PFS) compared to placebo in patients with stage III unresectable NSCLC [18]. Furthermore, there are several studies in progress to explore how to optimize the addition of immunotherapy to multimodality treatment in resectable NSCLC [19], such as IMpower010. This study showed that maintenance treatment with atezolizumab after adjuvant platinum-based chemotherapy significantly prolonged disease-free survival compared to adjuvant platinum-based chemotherapy alone in patients with resected stage II-IIIA NSCLC [20, 21].

Cancer cost the European Union €126 billion in 2009, and lung cancer had the highest economic cost (15% of overall cancer cost) [22]. Although the clinical burden of the disease is well known, there are few real-world data on the economic impact [23]. So, despite the high incidence and clinical burden of NSCLC, and the fact that relapse is the main cause of mortality during postresectional treatment, data on the cost of disease recurrence in Spain are scarce.

The aim of this study is to assess the economic burden of disease recurrence, estimating the cost of a locoregional or metastatic relapse after appropriate early-stage NSCLC treatment in Spain.

## Materials and methods

### Model design

A decision-tree model was developed to estimate the economic burden of a recurrence after appropriate treatment of early-stage NSCLC. The model was designed to calculate the costs associated with a locoregional or a metastatic recurrence:

- If the relapse is defined as locoregional (local or regional), the patient may be susceptible to local treatment with curative intent and thereafter a possibility of disease remission is considered. It was assumed that patients unsuitable for local treatment and patients who are not in remission, will eventually relapse and develop metastatic disease (second recurrence).
- In case of metastatic relapse, or in those patients with locoregional relapse who eventually progress, subsequent treatment lines are administered according to treatment response or until unacceptable toxicity. Treatments for metastatic disease vary according to histology (squamous or adenocarcinoma), biomarkers detected (*EGFR, ALK, ROS1*) and the level of PD-L1 expression, reflected in this the decision-tree model. Since there is no therapy reimbursed by the national health system (NHS) for *BRAF* mutated NSCLC patients in Spain, they were considered as wild-type (WT) patients for treatment purposes in the model.

Model results are expressed in total costs incurred per 100 NSCLC patients experiencing relapse. Mean cost of a locoregional or metastatic relapse are estimated as well. The time horizon for estimating the costs of locoregional relapse was one year, whereas for metastatic relapse it corresponds to the duration of the subsequent treatment lines received (patients who die out of the model).

The base case analysis was performed from the Spanish NHS perspective (including medical costs) and from a societal perspective (including indirect costs derived from the impact of the disease on labour productivity).

### Data collection

Since no published information of the healthcare resource consumption in Spain associated to NSCLC recurrence were found, a two-round consensus panel was conducted. The panel of experts was integrated by 8 Spanish clinical experts (4 oncologists and 4 hospital pharmacists) from different Spanish regions. In the first round, the 8 experts were asked to complete a questionnaire describing patient’s flow according to: relapse type (locoregional or metastatic); relapse characterization (histology, biomarker status); treatment pathways of both locoregional and metastatic recurrence (up to 4 lines characterized according to histology and biomarker status); use of healthcare resources; and sick leave due to locoregional or metastatic relapse.

Afterwards, mean responses were calculated, and a second round was carried out to share the answers of the first-round with the experts, aiming to reduce variability and to reach a common consensus in those variables where first-round consensus was not reached.

### Characterization of the metastases

In advanced or metastatic NSCLC, treatment selection is established according to the histological characteristics and molecular characterization of the patient. Therefore, the distribution of the NSCLC histologies and the prevalence of alterations in the main biomarkers were established by the panel of experts as follows: 30% squamous and 70% non-squamous (adenocarcinoma).

Among adenocarcinoma patients, the expert panel agreed upon the following prevalence of alterations: *ALK*-rearrangement (*ALK* +) in 3.4%, *EGFR*-mutation (*EGFR* +) in 13.6%, *ROS1*-mutation (*ROS1* +) in 2.0%. Among the WT patients (without alterations in the described biomarkers) the overexpression of PD-L1 was established according to tumor proportion score (TPS) > 50%, being overexpressed in 34.7% of adenocarcinoma patients.

### Direct medical costs (healthcare resource utilisation and unit costs)

The following direct medical costs were included in the model: drug acquisition costs, cost associated to treatment-related adverse events management, and healthcare resources costs such us local procedures (surgery, radiotherapy), hospitalizations, day hospital visits for drug administrations, follow-up visits, tests and imaging studies, etc.).

#### Relapse diagnosis

First, some healthcare resources are consumed to establish the type and characteristics of the relapse. The panel of experts considered that all patients need a complete blood test, 84% of the patients require a Positron emission tomography (PET)/Computed tomography (CT scan), 40% a Nuclear magnetic resonance (NMR), and other procedures such us ultrasound, scintigraphy or X-rays was required by less than 20% of patients.

If the relapse is metastatic, a patient's molecular profile study is needed to establish the corresponding treatment. If the molecular study was not performed at the initial diagnosis, or if a long time has passed since the initial diagnosis, it is necessary to re-biopsy the patient, and it has been established that this occurs in approximately half of the metastatic relapses. The procedure for re-biopsy established by the experts was: Fine-needle aspiration (FNA) 48.8%, core needle biopsy (CNB) 27.5%, liquid biopsy 18.8%, and bronchoscopy or endobronchial ultrasound (EBUS) 5%.

Regarding the molecular analysis of the patient, the techniques considered to detect genetic alterations were RT-PCR (for *EGFR*) and immunohistochemistry and fluorescent in-situ hybridization (FISH) for ALK and ROS1. Next-generation sequencing (NGS) panel was considered to be used in 20% of the patients and it covers the detection of all the biomarkers.

#### Treatment costs

**Fig. 1:**
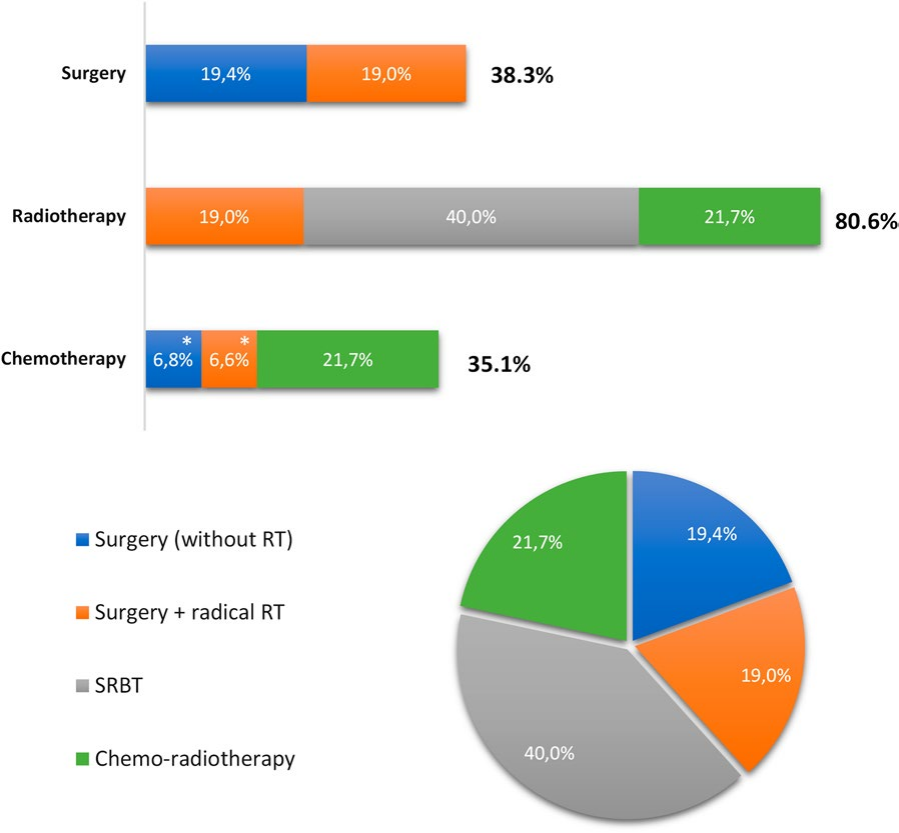
Local treatments in locoregional relapse. a shows the different local treatments grouped into surgery, radiotherapy, and chemotherapy, while 1b shows the distribution of these local treatments without grouping. *Adjuvant chemotherapy: 9% platinum + gemcitabine, 13% platinum + pemetrexed, 70% platinum + vinorelbine, 8% platinum + paclitaxel. **Chemo-radiotherapy: 38% platinum + vinorelbine, 31% platinum + paclitaxel, 31% platinum + etoposide

If the relapse is defined as locoregional, in candidates for local treatment, the expert panel considered that 38.3% undergo a surgical procedure (lobectomy), while 80.6% receive some kind of radiotherapy (RT). Specifically, 19.0% are treated with radical RT adjuvant or neoadjuvant to surgery, 40.0% with stereotactic body radiotherapy (SBRT), and 21.7% with chemotherapy plus concomitant radiotherapy (CT–RT). Also, 35.1% of the patients receive platinum-based chemotherapy, 21.7% as CT–RT (without surgery) and 13.4% as adjuvant to surgery. Among patients receiving CT–RT, those with stage III are candidates for durvalumab maintenance (approximately 45% according to the experts panel). Figure 1 depicts the local treatments distribution considered in the model in case of locoregional relapse.

If metastases are diagnosed at the time of relapse (or the disease has progressed from a previous locoregional relapse), the patient starts a first-line (1L) of oncologic treatment depending on the histology and molecular profile of the tumor. According to the consensus reached by the expert panel through the 2 rounds of questionnaires:

- 96.4% of squamous histology patients (30% of the total) receive a first-line treatment, and subsequently 52.4%, 24.5% and 4.0% reached second-line (2L), third-line (3L) and fourth-line (4L), respectively.
- 97.1% of adenocarcinoma histology patients (70% of total) receive a 1L treatment, and subsequently 61.5%, 34.9% and 13.1% reached 2L, 3L and 4L, respectively.

**Fig. 2:**
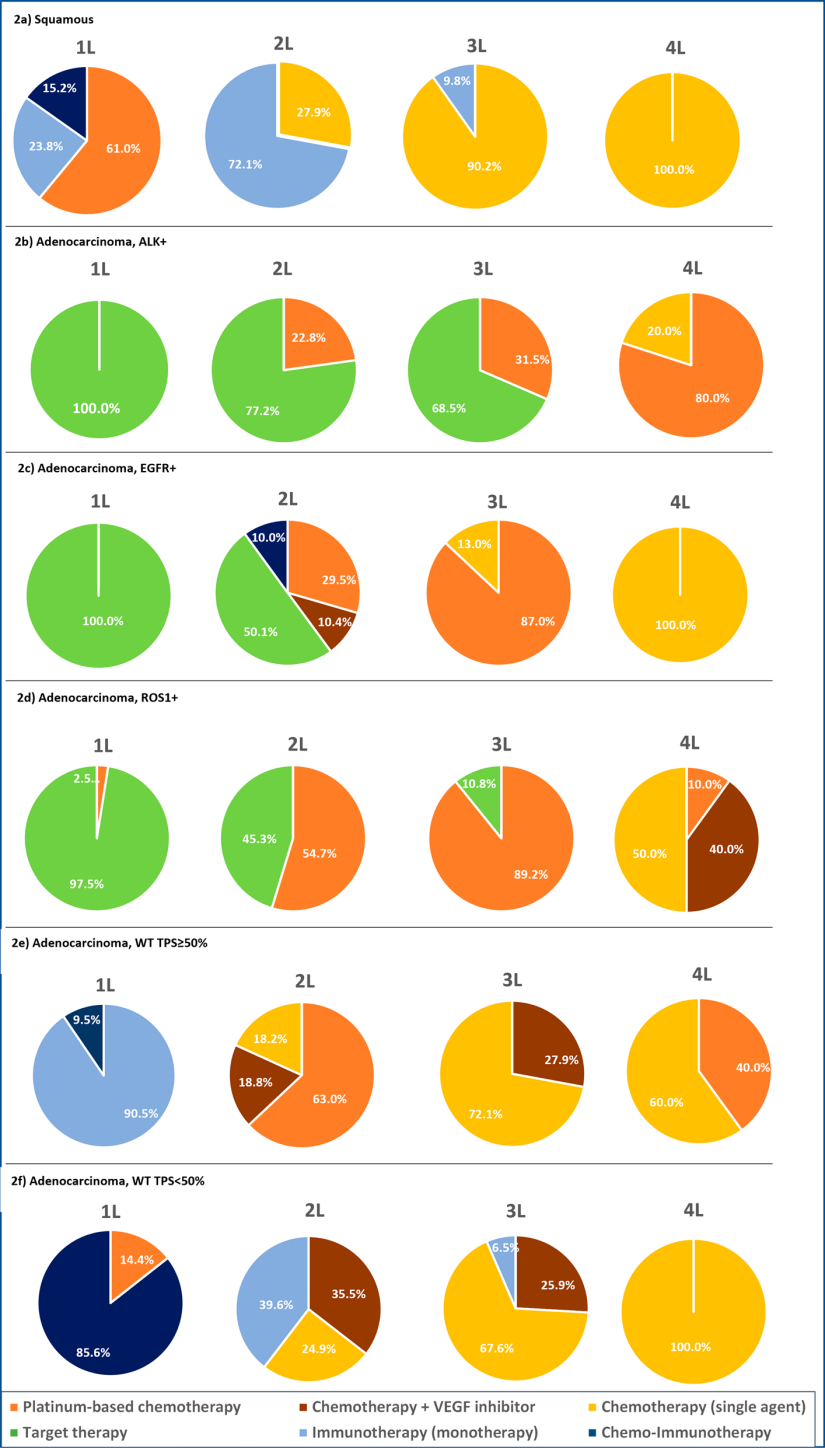
Pharmacological treatment distribution in each line of treatment after metastatic relapse. 1L: first-line; 2L: second-line; 3L: third-line; 4L; forth-line

The distribution of treatments in each line for the different histological subtypes and molecular profiles is shown in Fig. 2. Treatments are grouped into the following categories: platinum-based chemotherapies, chemotherapies plus VEGF inhibitors, chemotherapies with a single agent, targeted therapies with a TKI, immunotherapies as monotherapy, and chemo-immunotherapies. The detailed distribution with specific treatments is shown in Additional files 1, 2, 3, 4, 5, 6.

Posology for each specific treatment was obtained from their respective summary of product characteristic [24]. The duration of 1L and 2L treatments was estimated from the median PFS reported in the respective clinical trials [25–47], and it was assumed that patients reaching 3L and 4L receive approximately 4.5 cycles and 3 cycles, respectively. Finally, based on the distribution of treatments for each line and the median PFS for each treatment, the weighted mean of the time spent on each treatment line was estimated.

As a result, 1L average duration was estimated in 6.3 months for patients with squamous histology and 33.6, 18.2, 19.0, 8.4 and 10.2 months for *ALK* + , *EGFR* + , *ROS1* + , WT-TPS < 50% and WT-TPS > 50%patients, respectively. 2L average duration was 3.1 months for patients with squamous histology and 7.1, 7.5, 5.9, and 3.3 months for *ALK* + , *EGFR* + , *ROS1* + and WT patients, respectively. For all patients, 3L and 4L length were 3.1 and 2.1 months respectively.

#### Healthcare resource use

The consumption of all healthcare resources (expressed in the percentage of patients using the resource and the annual frequency) was obtained from the two-round consensus panel. Table 1 summarizes the use of resources in case of locoregional relapse or metastatic relapse (differentiated by treatment line).

**Table 1:** Healthcare resource use by percentage of patients using the resource (1a) and by annual frequency (1b)

	Locorregional relapse	Metastatic relapse			
		1L	2L	3L	4L +
*a Health resource (percentage of patients*					
ED visits	30.0%	38.6%	51.4%	61.4%	64.3%
Hospitalizations	17.1%	21.3%	35.0%	45.0%	55.7%
Visit to day hospital	55.7%	71.9%	85.7%	77.1%	65.7%
Specialist visits	100.0%	87.5%	100.0%	98.6%	97.1%
Laboratory analysis	100.0%	100.0%	100.0%	100.0%	100.0%
Bone scintigraphy	13.6%	20.0%	17.9%	19.3%	17.1%
PET/CT-scan	35.0%	11.4%	7.1%	5.7%	4.3%
Bone X-ray	27.3%	45.7%	52.9%	52.9%	58.6%
Nuclear magnetic resonance	43.6%	27.9%	34.3%	32.1%	27.1%
CT scan (Brain)	0.0%	14.3%	14.3%	14.3%	14.3%
CT scan (Others)	97.1%	99.3%	99.3%	99.3%	97.1%
*b) Health resource (annual frequency)*					
ED visits	2.0	2.4	2.9	3.6	3.7
Hospitalizations	5.3	5.1	6.6	7.6	8.6
Visit to day hospital (oral / IV treatments)	6.4 / 17.0	12.0/17.0	12.0/17.0	12.0 / 17.0	12.0 / 17.0
Specialist visits (oral / IV treatments)	6.4/ 17.0	12.0/17.0	12.0/17.0	12.0 / 17.0	12.0 / 17.0
Laboratory analysis (oral / IV treatments)	6.4/ 17.0	12.0/17.0	12.0/17.0	12.0/17.0	12.0/17.0
Bone scintigraphy	1.2	1.7	1.4	1.5	1.3
PET/CT-scan	1.0	0.7	0.7	0.6	0.4
Bone X-ray	2.6	2.4	2.9	2.6	3.2
Nuclear magnetic resonance	1.7	2.3	1.7	1.9	1.6
CT scan (Brain)	0.0	0.4	0.4	0.4	0.4
CT scan (Others)	3.7	4.1	4.1	3.9	4.0

*1L* first-line; *2L* second-line; *3L* third-line; *4L* forth-line; *PET/CT-scan* positron emission tomography/computed tomography; *ED* emergency department

The frequency of resource consumption in Table 1 is shown annualized to simplify interpretation, but in the model it has been adjusted for the average duration of each treatment line.

In case of metastases, in addition to the described disease management, there is an additional use of resources that varies depending on the metastasis location. According to the panel of experts, the most frequent distant disease localizations were bone metastases (47%), adrenal metastases (44%), lung metastases (40%), brain metastases (40%) and liver metastases (40%). Additional file 7 shows the specific procedures for each metastasis site and the percentage of patients using this resource.

Finally, treatment-related adverse events (AEs) were also included in the model. Grade ≥ 3 AEs reported with a frequency ≥ 5% in their respective clinical trials were considered [27, 30, 31, 33, 36, 38, 43, 45, 46, 48–57]. The most frequent AEs among treatments included in the model were neutropenia, anaemia, decreased white blood cell count and neutropenia.

#### Unit costs

Direct health costs were calculated by multiplying the natural units of the resources used described in the previous section by the corresponding unit cost. All the unit costs were obtained from national databases and are expressed in euros of 2022.

Pharmacological costs were expressed as the ex-factory price considering (when appropriate) the corresponding deductions according to Royal Decree Law 08/2010 [58, 59].

Healthcare unit costs were obtained from the Spanish healthcare database [60] and are summarized in Additional file 8.

### Indirect costs

Indirect costs measured by productivity losses due to sick leave were calculated using the human-capital approach, considering that the salary reflects the worker’s productivity [61]. It was estimated that 25% of patients were under 65 years old at the time of relapse and therefore of working age. Among the working-age population, the average annual salary for the 45–65 years old age range was obtained from the National Salary Structure Survey [62] and adjusted by the unemployment rate of 9.47% for that age range (INE).

The panel of experts agreed that due to relapses, 33% of patients who suffer a locoregional relapse return to work after an average sick leave of 7, 6 months, with the remaining 66% taking indefinite leave. All patients with metastatic relapses take indefinite sick leave and never return to work.

### Sensitivity analysis

Uncertainties were explored through a multi-way sensitivity analysis [63] which also provided a range to the mean values reported as base case. In the multi-way sensitivity analysis, the following model parameters were modified by a 20% increase or decrease from the baseline value: number of treatment cycles, location of the metastasis, percentage of patients using a healthcare resource, frequencies of healthcare resources, healthcare unit costs, patients of working age and average annual salary.

## Results

### Total cost per 100 relapses

**Fig. 3:**
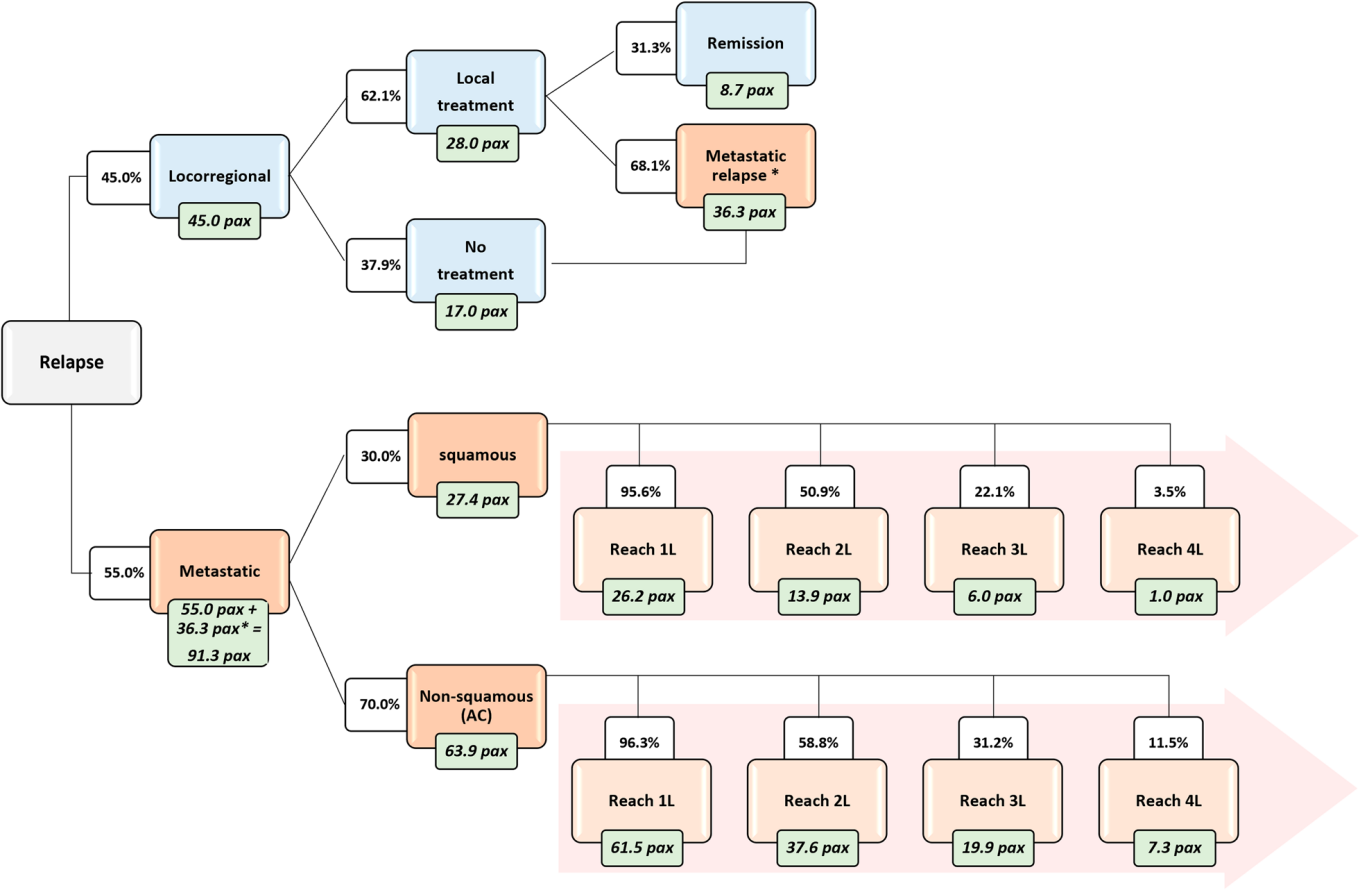
Patient’s flow. Patients with untreated or treated locoregional relapse who do not achieve remission (36.3 pax) are incorporated into the metastatic relapse arm, as the disease will eventually progress. Pax: patients; 1L: first-line; 2L: second-line; 3L: third-line; 4L; forth-line

The decision-tree model shows that in a 100-patients cohort experiencing a relapse, 45 would be diagnosed as locoregional relapse, of which 36.3 would eventually progress to metastasis and 8.7 patients would be in remission after local treatment with curative intent. Over time, 91.3 patients would experience a metastatic relapse in the 100-patients cohort, 55 as a first relapse and 36.3 after a previous locoregional relapse (Fig. 3).

Table 2 shows the overall cost of the 100-relapsing patient's cohort according to the Fig. 3 flowchart. Direct costs account for 78.0% and 93.5% of the total cost of locoregional and metastatic relapses, respectively.

**Table 2:** Main results of the cost analysis of 100 relapsing patients

	Total 100-patients cohort		
	Direct costs	Indirect costs	Total costs
**Relapse diagnosis**	**€98,020**	**n.a**	**€98,020**
**Locoregional relapse**	**€550,082**	**€154,915**	**€704,997**
**Metastatic relapse**	**€8,668,679**	**€604,149**	**€9,292,828**
Characterization of the metastasis	*€31,724*	*n.a*	*€31,724*
Distant metastases specific treatment	*€414,479*	*n.a*	*€414,479*
1L	*€7,171,435*	*€451,255*	*€7,622,691*
2L	*€816,838*	*€101,536*	*€918,375*
3L	*€196,215*	*€42,356*	*€238,571*
4L +	*€57,987*	*€9001*	*€66,988*
**Total cost**	**€9,336,782**	**€759,064**	**€10,095,846**

*1L* first-line; *2L* second-line; *3L* third-line; *4L* forth-line

In bold, main cost categories and final sum. In italics, subcategories of metastatic relapse

### Average relapse cost

**Fig. 4:**
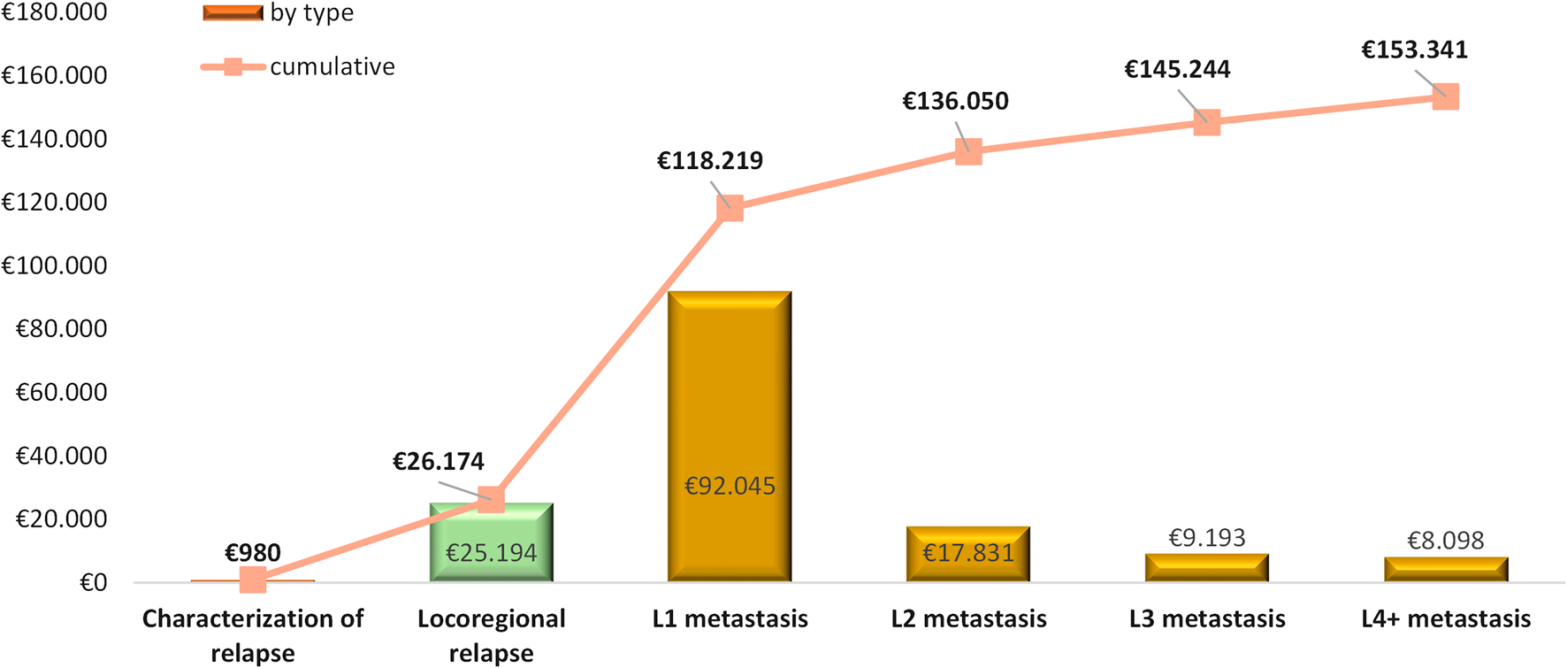
Per-patient average relapse cost by type of relapse and treatment line, and accumulative. Pax: patients; 1L: first-line; 2L: second-line; 3L: third-line; 4L; forth-line

The cost of one locoregional relapse was estimated in €25,194 (€19,658 direct costs, €5536 indirect costs), while the average cost of a metastatic relapse depends on the number of treatment lines received. The average cost of a patient with metastasis receiving only 1L treatment is €92,045 (€86,898 direct, €5148 indirect), whereas if the patient reaches the 4L the average cost (cumulative) is €127,167 (€117,328 direct, €9839 indirect). Figure 4 shows the average cost per-patient considering that a locoregional relapse is experienced and eventually there is metastatic recurrence.

In a metastatic relapse scenario, the histological and molecular profiles of the patients show different costs. The cost of an *ALK* + relapsed patient is the highest among metastatic treatments, with a direct cost of €202,203 for 1L, but its contribution to the average cost of metastatic relapse is moderate due to the low prevalence of *ALK* rearrangements (3, 4%). On the other hand, the lowest treatment cost is associated with squamous patients, with a 1L direct cost of €34,346 (Additional file 9).

### Sensitivity analysis results

The multi-way sensitivity analysis shows that, in proportion, more variability was observed for the indirect cost estimation than for the direct cost estimation. The mean cost of locoregional relapse was €25,194 (range €18,073–33,173), with €19,658 (range €14,437–25,405) of direct costs and €5536 (range €3636–7768) of indirect costs. Mean cost of metastatic relapse when 4L is reached was €127,167 (range €91,427–171,688) in overall, with €117,328 (range €86,389–154,685) of direct costs and €9839 (range €5038–17,002) of indirect costs.

## Discussion

Within the first 5 years after surgery, disease recurrences are the main cause of mortality during postresection treatment of early-stage NSCLC [16]. Despite the clinical relevance of postresectional NSCLC relapse, to our knowledge, this is the first study to date quantifying the global cost of a recurrence after early-stage NSCLC treatment in Spain. Our study was carried out through the consultation of an expert panel from different Spanish regions and takes into account the type of relapse and the number of treatment lines received (decision tree model). The Spanish NHS is based in the principles of universality, free access, equity and fairness of financing, and is mainly funded by taxes. It is organized at two levels—national and regional—mirroring the administrative division of the country, so the participation of experts from different regions was crucial [64].

By means of a two-round consensus validation and the elaboration of a decision-tree model, it was found that the average cost of a metastatic relapse is considerable (€127,167 if the patient receives up to 4 lines of treatment), mainly due to the cost associated with the 1L treatment, which represents the 68.4% of the average cost of all lines received in metastatic relapse. In recent years, several innovative therapies have been incorporated into the therapeutic landscape of NSCLC, such as TKIs targeting *ALK*, *EGFR*, or *ROS1* or immunotherapies (in monotherapy or in combination with chemotherapy) in WT patients [1, 9, 11, 12]. These new treatments have considerably increased the PFS of NSCLC patients, but at the same time have increased the cost of first lines treatments [20, 21].

The average cost of a locoregional recurrence (€25,194) was substantially lower than metastatic relapse. It is worth noting that even considering that only patients with stage III after concurrent chemoradiotherapy were candidates for maintenance treatment with durvalumab, the greater part of a locoregional relapse cost derives from the 1-year treatment with durvalumab (according to the PACIFIC trial scheme) and not from surgical procedures [18]. Therefore, it is expected that the cost of these non-metastatic relapses will increase with the approval of various immunotherapies in the adjuvant treatment of early stages.

In addition to the average cost of a locoregional or metastatic relapse, our analysis estimates the total cost for a cohort of 100 relapsing patients, which measures the occurring relapses by type. By showing the results per 100 relapses, the differences between the cost of locoregional relapses and metastatic relapses are more pronounced than with the average cost of 1 relapse. This is because even if a patient experiences a locorregional relapse, they eventually end up developing metastases, and only one-third of the patients achieve disease remission and can be considered as 'cured' after appropriate treatment of locorregional relapse. Therefore, the number of metastatic relapses is considerably higher than locoregional relapses when a cohort of 100 patients is analysed.

At the national level, some studies analysed the costs of diagnosis and treatment of many cancers, including lung cancer. A retrospective observational design study between 2010 and 2015 estimated the mean 3-year costs per patient with stage I to III lung cancer in Spain in €12,023; also, total survival-adjusted costs until death for patients with stage IV disease was €16,151 [65]. Focusing on the mean cost per patient with NSCLC diagnosed and treated in hospitals form Catalonia, the cost estimated by Corral et al. ranged from €13,218 (stage III) to €16,120 (stage II) in a retrospective, descriptive analysis on resource use and a direct medical cost analysis carried out in a 197-patient cohort with NSCLC [66]. Additionally, treatment patterns, use of resources and costs associated with treating advanced or metastatic NSCLC patients in Spain have been described through a Delphi panel methodology, estimating a total cost per patient with advanced or metastatic NSCLC in €11,301 and €32,754 depending on the number of treatment lines received for metastatic NSCLC patients [67].

Regarding the specific costs of diagnosis and treatment of NSCLC early-stages, Andreas et al. estimated the burden and cost-of-illness for 306 patients with completely resected stage IB-IIIA NSCLC in France, Germany and the United Kingdom (UK). The mean total direct costs per patient during the follow-up period were €19,057 in France, €14,185 in Germany and €8377 in the UK, whereas mean total indirect costs per patient were estimated in €696, €2476, and €1414 for France, Germany and the UK respectively [68]. Moreover, a recent retrospective chart review study collecting data from 2L + patients with advanced NSCLC (973 patients) in some European countries estimated a mean patient cost of € 24,414 in a cohort of 200 Spanish patients [23]. A recent Italian study developed a detailed “whole‐disease” model listing the probabilities of all potentially necessary diagnostic and therapeutic actions involved in the management of each stage of NSCLC. In this study the NSCLC patient cost was estimated in €16,291 in stage I, €19,530 in stage II, €21,938 in stage III and €28,711 in stage IV [69]. As several authors point out, in the early stages of the disease, the main cost is incurred by surgery, whereas in the more advanced stages the cost of chemotherapy and adjuvant therapy becomes more relevant [65, 66, 68, 69].

All the studies mentioned above present methodological differences with our model making the comparison of results difficult. On the one hand, several of the studies discussed are based on prospective or retrospective data. On the other hand, studies such as the one by Buja et al. using a model to calculate the cost associated with the treatment of metastatic NSCLC use 1-year time horizons and do not include successive treatment lines. In fact, no studies were identified that specifically estimated the cost of relapse in NSCLC. However, in other cancer research areas, we found publications following similar methodologies to ours, such as the work carried out by Albanell et al. [70] that estimated the costs of recurrence in patients with *HER2* + breast cancer in Spain.

Although the sensitivity analyses performed show that the results are robust, our study is not exempt of some limitations. One of these is inherent to theoretical models, whose structural rigidity prevents a comprehensive representation of routine clinical practice due to the qualitative methodology used to estimate resource utilization associated with NSCLC relapses. Another limitation arises from data gathering, which was carried out through a two-round consensus panel of 8 Spanish clinical experts and not collected in prospective or retrospective studies. In addition, the panel of experts can be considered small in comparison to a Delphi panel, so it may not be fully representative of the whole country. A third limitation derives from the time horizon and the number of treatment lines included in the model. In metastatic relapses, the duration of treatment lines (estimated by median PFS), is highly variable depending on the histology and molecular profile of the patient, exceeding one year in several first-line treatments. In locoregional relapse, durvalumab in maintenance for 1 year is the only long-term treatment (adjuvant chemotherapies and radiotherapies usually last no more than 3 months), so the resource use estimation associated with locoregional relapse was limited to 1 year. The European Society for Medical Oncology (ESMO) guidelines recommends surveillance every 6 months for 2 years and thereafter an annual visit after treatment with curative intent in early-stages NSCLC patients [71], so the estimation of a locorregional recurrence could be underestimated in our model.

Regarding the treatment costs, which represent a large share of the total cost (especially in the metastatic settings), the enrolment of relapsing patients in clinical trials was not considered. This aspect represents relevant savings for hospitals given that pharmaceutical company covers the cost of treatment [72–74], therefore, our results may be slightly overestimated. Furthermore, our model did not consider dose reductions, which might again overestimate the cost of active treatment, although, on the other hand, full vial sharing was considered for IV treatments cost calculation (no wastage), therefore underestimating treatment costs.

With respect to the indirect cost estimation, a lifetime time horizon for relapsing patients and caregiver costs was not considered, so from the societal perspective, the cost of relapsing patients could have been underestimated.

As occurs with all cost-of-illness analyses, drug costs and unit costs of healthcare resources can vary considerably between countries. Also, patient management and resource consumption can markedly differ between countries and centers in the same country. Therefore, future research with a prospective methodological design would be desirable to accurately quantify the healthcare resource consumption in relapsing patients with NSCLC. Lastly, it is worth mentioning that the analysis was conducted based on the clinical experience of a group of Spanish experts, mainly from first-level hospitals, so caution should be exerted when transferring results.

## Conclusion

Our study provides an estimation of the specific cost of a relapse in patients diagnosed with early-stage NSCLC who have received the appropriate treatment with curative intent. We focused on estimating the direct and indirect costs in locoregional and metastatic relapses considering that successive treatment lines are received. Our findings suggest that the overall cost of a relapse is substantial, and it increases considerably in the metastatic relapse setting, mainly due to the high cost and long duration of 1L treatments. Therefore, we believe that in addition to new treatments for metastatic disease, novel treatment options that further reduce the risk of recurrence after treatment of early-stage NSCLC are also needed.

## Supplementary Information

**Additional file 1:** Treatment distribution in squamous patients.

**Additional file 2:** Treatment distribution in ALK+ adenocarcinoma patients.

**Additional file 3:** Treatment distribution in EGFR+ adenocarcinoma patients.

**Additional file 4:** Treatment distribution in ROS1+ adenocarcinoma patients.

**Additional file 5:** Treatment distribution in WT PD-L1+, TPS ≤ 50% adenocarcinoma patients.

**Additional file 6:** Treatment distribution in WT PD-L1+, TPS > 50% adenocarcinoma patients.

**Additional file 7:** Health resources consumption in specific distant metastases.

**Additional file 8:** Healthcare unit costs.

**Additional file 9:** Cost of a metastatic relapse by molecular profile.

## Data Availability

Qualified researchers may request access to individual patient level data through the clinical study data request platform (https://vivli.org/). Further details on Roche’s criteria for eligible studies are available here (https://vivli.org/members/ourmembers/). For further details on Roche’s Global Policy on the Sharing of Clinical Information and how to request access to related clinical study documents, see here (https:// www.roche.com/ research_ and_ development/ who_ we_ are_ how_ we_ work/ clinical_ trials/ our_ commitment_ to_data_ sharing. htm).

## Abbreviations

1L: First-line
2L: Second-line
3L: Third-line
4L: Fourth-line
AE: Adverse event
CNB: Core needle biopsy
CT: Chemotherapy
EBUS: Endobronchial ultrasound
ED: Emergency department
ESMO: European Society for Medical Oncology
FISH: Fluorescent in-situ hybridization
FNA: Fine-needle aspiration
NGS: Next-generation sequencing
NHS: National health system
NMR: Nuclear magnetic resonance
NSCLC: Non-small cell lung cancer
OS: Overall survival
PET: Positron emission tomography
PFS: Progression-free survival
SBRT: Stereotactic body radiotherapy
SEER: Surveillance, epidemiology, and end results
SEOM: Spanish Society of Medical Oncology
RT: Radiotherapy
TPS: Tumor proportion score
WT: Wild type
