# Supplementary material for: Economic burden of locoregional and metastatic relapses in resectable early-stage non-small cell lung cancer in Spain
Source: BMC Pulm Med. 2023 Feb 21;23:69. doi: 10.1186/s12890-023-02356-0 (PMC9942326; doi:10.1186/s12890-023-02356-0)
Supplement: Supplementary file 1 — Additional file 1: Treatment distribution in squamous patients. [file 12890_2023_2356_MOESM1_ESM.docx]

**Additional File 1**. Treatment distribution in squamous patients

| **1L** | **%** |  |
| --- | --- | --- |
|  |  |  |
| Platinum + gemcitabine | 22.9% |  |
| Platinum + pembrolizumab + paclitaxel | 15.2% |  |
| Pembrolizumab | 23.8% |  |
| Platinum + paclitaxel | 24.2% |  |
| Platinum + vinorelbine | 13.9% |  |
| **2L** | **%** |  |
| Atezolizumab | 22.0% |  |
| Nivolumab | 36.2% |  |
| Pembrolizumab | 13.9% |  |
| Docetaxel | 27.9% |  |
| **3L** | **%** |  |
| Docetaxel | 54.4% |  |
| Atezolizumab | 9.8% |  |
| Vinorelbine | 18.9% |  |
| Gemcitabine | 16.9% |  |
| **4L+** | **%** |  |
| Vinorelbine | 60.0% |  |
| Gemcitabine | 40.0% |  |

*1L: first-line; 2L: second-line; 3L: third-line; 4L; forth-line*
