# Supplementary material for: Economic burden of locoregional and metastatic relapses in resectable early-stage non-small cell lung cancer in Spain
Source: BMC Pulm Med. 2023 Feb 21;23:69. doi: 10.1186/s12890-023-02356-0 (PMC9942326; doi:10.1186/s12890-023-02356-0)
Supplement: Supplementary file 2 — Additional file 2: Treatment distribution in ALK+ adenocarcinoma patients. [file 12890_2023_2356_MOESM2_ESM.docx]

**Additional File 2**. Treatment distribution in *ALK*+ adenocarcinoma patients

| **1L** | **%** |  |
| --- | --- | --- |
|  |  |  |
| Alectinib | 89.1% |  |
| Brigatinib | 10.9% |  |
| **2L** | **%** |  |
| Alectinib | 28.0% |  |
| Platinum + pemetrexed | 22.8% |  |
| Lorlatinib | 49.2% |  |
| **3L** | **%** |  |
| Lorlatinib | 68.5% |  |
| Platinum + pemetrexed | 31.5% |  |
| **4L+** | **%** |  |
| Platinum + pemetrexed | 80.0% |  |
| Docetaxel | 20.0% |  |

*1L: first-line; 2L: second-line; 3L: third-line; 4L; forth-line*
