# Supplementary material for: Economic burden of locoregional and metastatic relapses in resectable early-stage non-small cell lung cancer in Spain
Source: BMC Pulm Med. 2023 Feb 21;23:69. doi: 10.1186/s12890-023-02356-0 (PMC9942326; doi:10.1186/s12890-023-02356-0)
Supplement: Supplementary file 3 — Additional file 3: Treatment distribution in EGFR+ adenocarcinoma patients. [file 12890_2023_2356_MOESM3_ESM.docx]

**Additional File 3.** Treatment distribution in *EGRF*+ adenocarcinoma patients

| **1L** | **%** |  |
| --- | --- | --- |
|  |  |  |
| Osimertinib | 91.8% |  |
| Afatinib | 3.4% |  |
| Erlotinib | 4.8% |  |
| **2L** | **%** |  |
| Osimertinib | 50.1% |  |
| Platinum + paclitaxel+ bevacizumab | 10.4% |  |
| Platinum + pemetrexed | 29.5% |  |
| Platinum+paclitaxel+bevacizumab+atezolizumab | 10.0% |  |
| **3L** | **%** |  |
| Platinum + pemetrexed | 87.0% |  |
| Docetaxel | 13.0% |  |
| **4L+** | **%** |  |
| Gemcitabine | 72.5% |  |
| Docetaxel | 27.5% |  |

*1L: first-line; 2L: second-line; 3L: third-line; 4L; forth-line*
