# Supplementary material for: Economic burden of locoregional and metastatic relapses in resectable early-stage non-small cell lung cancer in Spain
Source: BMC Pulm Med. 2023 Feb 21;23:69. doi: 10.1186/s12890-023-02356-0 (PMC9942326; doi:10.1186/s12890-023-02356-0)
Supplement: Supplementary file 4 — Additional file 4: Treatment distribution in ROS1+ adenocarcinoma patients. [file 12890_2023_2356_MOESM4_ESM.docx]

**Additional File 4**. Treatment distribution in *ROS1*+ adenocarcinoma patients

| **1L** | **%** |  |
| --- | --- | --- |
|  |  |  |
| Crizotinib | 97.5% |  |
| Platinum + pemetrexed | 2.5% |  |
| **2L** | **%** |  |
| Platinum + pemetrexed | 19.0% |  |
| Entrectinib | 7.2% |  |
| Platinum + paclitaxel | 35.7% |  |
| Lorlatinib | 38.1% |  |
| **3L** | **%** |  |
| Platinum + paclitaxel | 50.0% |  |
| Platinum + pemetrexed | 39.2% |  |
| Lorlatinib | 10.8% |  |
| **4L+** | **%** |  |
| Docetaxel+ nintedanib | 40.0% |  |
| Docetaxel | 50.0% |  |
| Platinum + pemetrexed | 10.0% |  |

*1L: first-line; 2L: second-line; 3L: third-line; 4L; forth-line*
