# Supplementary material for: Economic burden of locoregional and metastatic relapses in resectable early-stage non-small cell lung cancer in Spain
Source: BMC Pulm Med. 2023 Feb 21;23:69. doi: 10.1186/s12890-023-02356-0 (PMC9942326; doi:10.1186/s12890-023-02356-0)
Supplement: Supplementary file 5 — Additional file 5: Treatment distribution in WT PD-L1+, TPS ≤ 50% adenocarcinoma patients. [file 12890_2023_2356_MOESM5_ESM.docx]

**Additional File 5.** Treatment distribution in WT PD-L1+, TPS≤50% adenocarcinoma patients

| **1L** | **%** |  |
| --- | --- | --- |
|  |  |  |
| Platinum + pemetrexed | 14.4% |  |
| Platinum+atezolizumab+bevacizumab+paclitaxel | 7.7% |  |
| Platinum + pemetrexed + pembrolizumab | 77.9% |  |
| **2L** | **%** |  |
| Nivolumab | 12.9% |  |
| Atezolizumab | 14.1% |  |
| Pembrolizumab | 12.6% |  |
| Docetaxel | 24.9% |  |
| Docetaxel+ nintedanib | 35.5% |  |
| **3L** | **%** |  |
| Docetaxel | 35.6% |  |
| Docetaxel+ nintedanib | 25.9% |  |
| Atezolizumab | 6.5% |  |
| Vinorelbine | 25.1% |  |
| Gemcitabine | 6.9% |  |
| **4L+** | **%** |  |
| Gemcitabine | 60.0% |  |
| Vinorelbine | 40.0% |  |

*1L: first-line; 2L: second-line; 3L: third-line; 4L; forth-line*
