# Supplementary material for: Economic burden of locoregional and metastatic relapses in resectable early-stage non-small cell lung cancer in Spain
Source: BMC Pulm Med. 2023 Feb 21;23:69. doi: 10.1186/s12890-023-02356-0 (PMC9942326; doi:10.1186/s12890-023-02356-0)
Supplement: Supplementary file 7 — Additional file 7: Health resources consumption in specific distant metastases. [file 12890_2023_2356_MOESM7_ESM.docx]

**Additional File 7.** Health resources consumption in specific distant metastases

| **Health resource** | **Percentage of patients** |  |
| --- | --- | --- |
|  |  |  |
| **Bone metastasis** | |  |
| Biophosphonates | 33.0% |  |
| Denosumab | 2.0% |  |
| Radiotherapy | 45.0% |  |
| Surgery (osteotomy) | 4.0% |  |
| Opioid analgesia | 14.0% |  |
| **Liver metastases** | |  |
| SBRT | 2.0% |  |
| Surgery (metastasectomy) | 8.0% |  |
| **Brain metastases** | |  |
| Radiotherapy | 46.0% |  |
| SBRT | 26.0% |  |
| Cirugía (brain metastasectomy) | 9.0% |  |
| **Adrenal metastases** | |  |
| SBRT | 12.0% |  |
| Cirugía (metastasectomy) | 17.0% |  |
