# Supplementary material for: Economic burden of locoregional and metastatic relapses in resectable early-stage non-small cell lung cancer in Spain
Source: BMC Pulm Med. 2023 Feb 21;23:69. doi: 10.1186/s12890-023-02356-0 (PMC9942326; doi:10.1186/s12890-023-02356-0)
Supplement: Supplementary file 8 — Additional file 8: Healthcare unit costs. [file 12890_2023_2356_MOESM8_ESM.docx]

**Additional File 8.** Healthcare unit cost

|  | **Unit cost** |  |
| --- | --- | --- |
|  |  |  |
| Surgery (lobectomy) | €1,520.5 |  |
| Surgery (osteotomy) | €1,675.1 |  |
| Surgery (brain metastasectomy) | €4,368.5 |  |
| Radical radiotherapy | €6,795.4 |  |
| SBRT | €6,420.6 |  |
| ED visits | €249.0 |  |
| Hospitalizations | €550.4 |  |
| Visit to day hospital (IV infusion) | €214.4 |  |
| Visit to day hospital (treatment collection) | €54.2 |  |
| Specialist visits | €88.4 |  |
| Laboratory analysis | €131.8 |  |
| Bone scintigraphy | €196.2 |  |
| PET/CT-scan | €741.6 |  |
| Bone X-ray | €42.9 |  |
| Nuclear magnetic resonance | €318.5 |  |
| CT-scan (Brain) | €192.4 |  |
| CT-scan (Others) | €169.4 |  |
| Panel NGS | €610.7 |  |
| RT-PCR | €119.5 |  |
| Inmunohistoquímica | €123.5 |  |
| FISH | €247.2 |  |
| Opioid analgesia | €824.4 |  |

*PET/CT-scan; positron emission tomography / computed tomography; ED: emergency department*
