# Supplementary material for: Economic burden of locoregional and metastatic relapses in resectable early-stage non-small cell lung cancer in Spain
Source: BMC Pulm Med. 2023 Feb 21;23:69. doi: 10.1186/s12890-023-02356-0 (PMC9942326; doi:10.1186/s12890-023-02356-0)

**Additional File 9.** Cost of a metastatic relapse by molecular profile

*1L: first-line; 2L: second-line; 3L: third-line; 4L; forth-line*


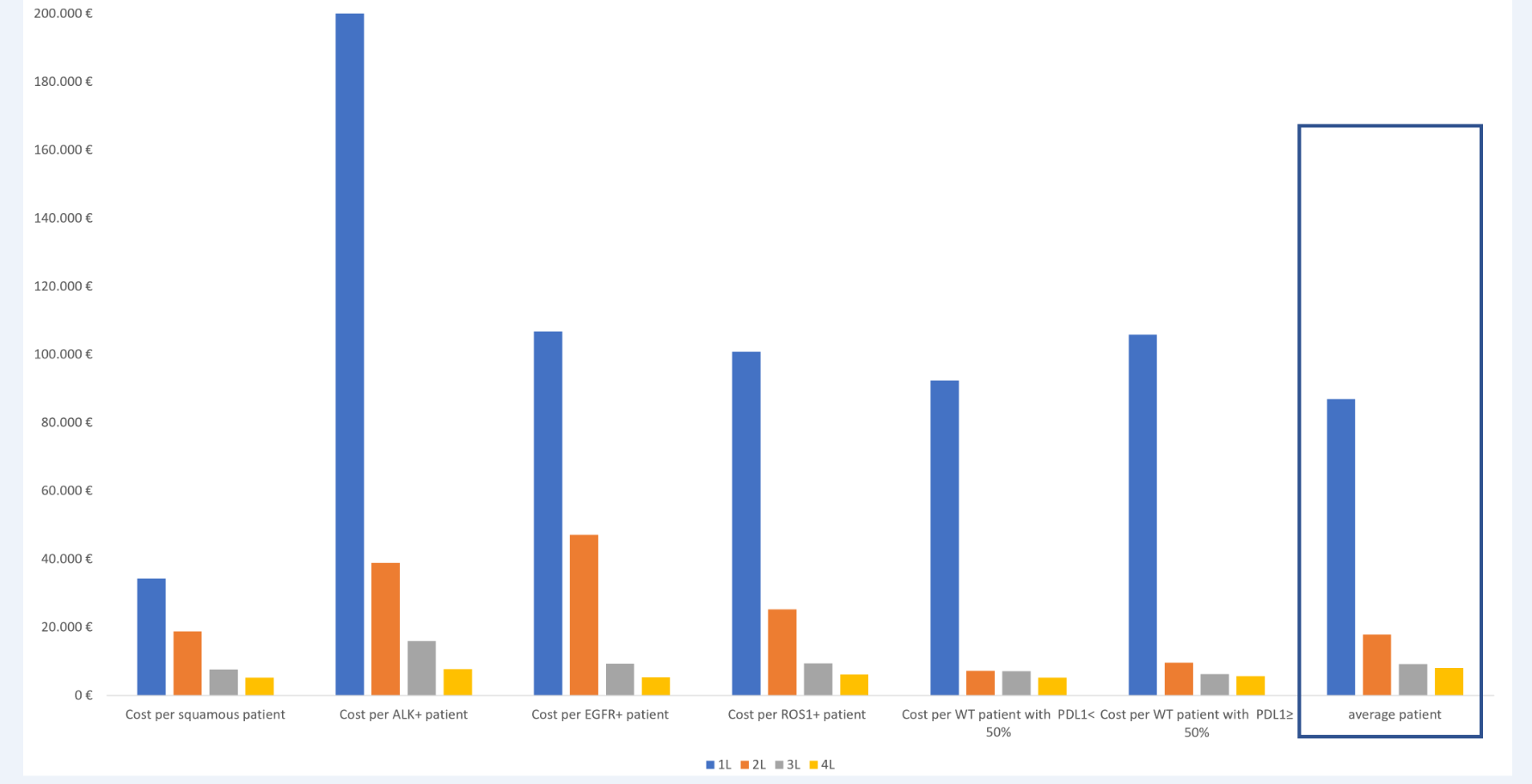

Supplement: Supplementary file 9 — Additional file 9: Cost of a metastatic relapse by molecular profile. [file 12890_2023_2356_MOESM9_ESM.docx]
